# Use of generalised additive models to categorise continuous variables in clinical prediction

**DOI:** 10.1186/1471-2288-13-83

**Published:** 2013-06-26

**Authors:** Irantzu Barrio, Inmaculada Arostegui, José M Quintana, IRYSS-COPD Group

**Affiliations:** 1 Departamento de Matemática Aplicada y Estadística e Investigación Operativa, Universidad del País Vasco UPV/EHU, Leioa, Spain; 2 Unidad de Investigación, Hospital Galdakao-Usansolo, Galdakao, Spain; 3 Red de Investigación en Servicios de Salud en Enfermedades Crónicas - REDISSEC, Galdakao, Spain

## Abstract

**Background:**

In medical practice many, essentially continuous, clinical parameters tend to be categorised by physicians for ease of decision-making. Indeed, categorisation is a common practice both in medical research and in the development of clinical prediction rules, particularly where the ensuing models are to be applied in daily clinical practice to support clinicians in the decision-making process. Since the number of categories into which a continuous predictor must be categorised depends partly on the relationship between the predictor and the outcome, the need for more than two categories must be borne in mind.

**Methods:**

We propose a categorisation methodology for clinical-prediction models, using Generalised Additive Models (GAMs) with P-spline smoothers to determine the relationship between the continuous predictor and the outcome. The proposed method consists of creating at least one average-risk category along with high- and low-risk categories based on the GAM smooth function. We applied this methodology to a prospective cohort of patients with exacerbated chronic obstructive pulmonary disease. The predictors selected were respiratory rate and partial pressure of carbon dioxide in the blood (PCO2), and the response variable was poor evolution. An additive logistic regression model was used to show the relationship between the covariates and the dichotomous response variable. The proposed categorisation was compared to the continuous predictor as the best option, using the AIC and AUC evaluation parameters. The sample was divided into a derivation (60%) and validation (40%) samples. The first was used to obtain the cut points while the second was used to validate the proposed methodology.

**Results:**

The three-category proposal for the respiratory rate was ≤ 20;(20,24];> 24, for which the following values were obtained: AIC=314.5 and AUC=0.638. The respective values for the continuous predictor were AIC=317.1 and AUC=0.634, with no statistically significant differences being found between the two AUCs (*p* =0.079). The four-category proposal for PCO2 was ≤ 43;(43,52];(52,65];> 65, for which the following values were obtained: AIC=258.1 and AUC=0.81. No statistically significant differences were found between the AUC of the four-category option and that of the continuous predictor, which yielded an AIC of 250.3 and an AUC of 0.825 (*p* =0.115).

**Conclusions:**

Our proposed method provides clinicians with the number and location of cut points for categorising variables, and performs as successfully as the original continuous predictor when it comes to developing clinical prediction rules.

## Background

Generally speaking, in medical practice it is quite common for physicians to take many clinical parameters that are essentially continuous variables and categorise them for practical reasons. Clinical practice tends to perceive test results as either normal or abnormal, or as normal, uncertain or abnormal, or alternatively to use such results as a severity scale of the patient’s prognosis for a given outcome. In particular, this is the case in clinical research where many continuous variables are categorised on the basis of the normality/abnormality principle, disease severity (ordinal) or grouping (non-ordinal). This practice reflects physicians’ way of thinking and their clinical decision-making process, and so in a research context categorisation is a common way [1] of rendering results more useful and more practical.

More specifically, a field where categorisation is widely used is that of clinical prediction rules (CPRs), which are relevant for decision-making in medicine [2]. Many CPRs are based on statistical models in which a number of clinical variables are regarded as potential predictors. Where the aim is to apply the model to routine clinical practice, definitions of outcomes and predictors should be in line with standard practice [2]. Furthermore, both the association between the predictor and the response variable, and the probability distribution of the predictor could determine the strength of the covariate in the model. Hence, the final model’s performance and goodness-of-fit will depend on the choice of a good and practical definition of the variables, an adequate measure of the association between predictor and outcome, and the strength of the covariate in the model. It is well documented that, from a purely statistical point of view, it is preferable to develop a statistical model using continuous rather than categorical covariates, since categorisation may lead to loss of information and reduction in power [3,4]. From a practical standpoint, however, continuous predictors are not often considered in the development of CPRs [2,5,6]. There are two main reasons for this: firstly, whenever a continuous covariate is incorporated into a model, the assumption of the linear relationship between the outcome and the covariate should be tested, and this is often not acceptable. Consequently, the statistical analysis itself could become a methodological challenge. Secondly, categorisation is like a mirror of clinical practice. Research results are important in the development of clinical practice, where decisions tend to be categorical in nature. Consequently, models with categorical predictors could be more easily understood and applied to clinical decision-making by clinicians. At times, decisions are based on clinical criteria that take a continuous marker as baseline, and select one or more cut points to determine whether or not a given patient has a certain status or diagnosis. Such criteria can vary enormously from one practitioner, hospital or even country, to another (an illustration of this will be given below); indeed, a meta-analysis conducted by Lim and Kelly showed that reported cut-off values for PCO2 (partial pressure of carbon dioxide in the blood) for screening hypercapnia ranged from 30 to 46 mmHg [7]. The search for adequate categorisation of clinical continuous variables is thus a relevant topic in the development of CPRs.

Previous work has been done on the categorisation of continuous variables. In a review of methods for categorising a predictor, the methods concerned were divided into two groups [6]: a) exploratory plots; and, b) a minimum p-value approach. Insofar as exploratory plots are concerned, Hin et al. proposed a method for dichotomising continuous variables using Generalised Additive Models (GAMs) [8]. The number of categories into which the continuous predictor should be categorised depends partly on the relationship -graphical and numerical- between the predictor and the outcome: hence, the need to create more than two categories should be borne in mind. This happens, for instance, with variables such as blood pressure, where a single cut point cannot be used to divide patients into high- and low-risk groups, since patients with high and those with low values are both associated with higher risk [9]. Furthermore, a very recent work criticised the arbitrary and frequent use made of quantiles in epidemiological research to categorise continuous variables [10].

Recent literature is unanimous in making the point that categorising continuous predictors should be well justified and motivated, and that, if necessary, the selected criterion for categorisation should be objective and sufficiently validated. The aim of this study was to propose a criterion for categorising continuous variables to be used in CPRs, by seeking both the optimal number of categories and the optimal cut points. The starting point of our proposed method is to examine the association between a continuous covariate and a pre-defined relevant outcome, using GAMs. This will allow us, in turn, to focus on the prediction of the chosen outcome, select the pertinent cut points, and create as many categories as required. The proposed method of categorisation considers the functional relationship between the covariate and the outcome, thereby ensuring that critical information is not lost from the continuous variable. Moreover, it is intended that the ensuing categorisation will be useful from a practical point of view, by combining good prediction ability with ease of use in routine clinical practice. The proposed method of categorisation was applied to real data drawn from the IRYSS-COPD Study (*IRYSS: Red de investigación cooperativa para la Investigación en Resultados de Salud y Servicios Sanitarios* - Co-operative Health Outcomes & Health Services Research Network), and then validated by comparing it to other methods.

The remainder of this paper is divided into three sections. In the Methods section, the GAM theory is briefly described and the proposed categorisation methodology is explained in detail. In addition, this section presents the IRYSS-COPD Study in which the proposed methodology is to be tested and validated, with a detailed outline of the methods used for validation purposes. The Results section reports the results of the application of the proposed methodology to real data as well as the validation process. Finally, the paper ends with a discussion and some conclusions.

## Methods

The Methods section is divided into two subsections: the first is devoted to the theoretical aspects of the methodology and includes a brief introduction to GAMs, in order to render the proposed methodology more easily understandable; the second focuses on the methods for applying the proposed methodology to real data and the criteria selected for validating the proposal.

### Theoretical methods

#### ***Generalised additive models***

GAM [11] is an extension of the Generalised Linear Model (GLM) where the modelling of the mean functions relaxes the assumption of linearity, albeit additivity of the mean function pertaining to the covariates are assumed. Whilst the mean functions of some covariates may be assumed to be linear, the non-linear mean functions are modelled using smoothing methods, such as kernel smoothers, lowess, smoothing splines or regression splines. In general, the model has the following structure

(1) $$\begin{array}{llll} g(\mu )=\alpha_{0}+\overset{p}{\underset{i=1}{\sum }}\;f_{i}\;(X_{i}) & & & \end{array}$$

where *μ* = *E*(*Y*) for *Y*, a response variable with some exponential family distribution, *g* is the *link* function, and *f*_*i*_ are some smooth functions of the covariates *X*_*i*_ for each *i* = 1,…,*p*.

GAMs provide more flexibility than do GLMs, as they relax the hypothesis of linear dependence between the covariates and the expected value of the response variable. The main drawback of GAMs lies in the estimation of the smooth functions *f*_*i*_, and there are different ways to address this. One of the most common alternatives is based on splines, which allow the GAM estimation to be reduced to the GLM context [12]. Splines are piecewise polynomials that join at points called knots: two major families belong to these models, i.e., regression splines and smoothing splines. Regression splines consist of selecting the number and location of the knots, and imposing restrictions so that the piecewise polynomials join smoothly. Smoothing splines [13], use as many knots as unique values of the covariate *X*_*i*_ and control the model’s smoothness by adding a penalty to the least-squares fitting objective [14]. An intermediate alternative to building the smooth functions -and one that considers the advantages of both smoothing and regression splines- is the use of penalised splines (also known as P-splines), introduced by Eilers and Marx [15]. P-splines use fewer knots than smoothing splines and introduce more general roughness penalties which relaxes the importance of the knot location. We have included a brief description of splines here and more detailed theoretical information is included in Appendix Appendix 1: Splines.

#### ***Categorisation methodology***

Our proposal consists of categorising continuous variables by using GAM models with P-spline smoothers. Without loss of generality, let us assume that there is a continuous variable X which we wish to categorise and a response variable Y with some exponential family distribution. In such a case, the GAM model defined in (1) is fitted with *X* as the covariate and *Y* as the response variable.

(2) $$\begin{array}{llll} g(\mu )=\alpha_{0}+f(X) & & & \end{array}$$

where *g* is the *link* function and *μ* = *E*(*Y*).

The aim of this method is to categorise the covariate *X*, based on the influence it has on the response variable *Y*. The number of categories as well as the location of the cut points will depend on the graphical relationship obtained by using the GAM model with P-spline smoothers. On the basis of this model, the graphical display shows the relationship between *X* and *f*(*X*), where *X* is plotted on the horizontal axis and the smooth function *f* is plotted on the vertical axis. *f*(*X*) is the centered mean function where the centering coefficient is *α*_0_, this is *f*(*X*) = 0 refers to the average value of the covariate. We therefore propose to start by creating an average-risk category around this average-risk point, together with as many high- and low-risk categories as are required to capture the relationship between *X* and *f*(*X*), as outlined in detail below.

We consider an average-risk category, by building an interval around the point *x*_0_ ∈ *X*, such that *f*(*x*_0_) = 0. To do so, we calculate the value for *x*_0_, by computing the inverse of *f*, and then the estimated value $\hat{\mu_{0}}$, such that:

$$\hat{\mu_{0}}=g^{-1}(\alpha_{0}+f(x_{0}))=g^{-1}(\alpha_{0})$$

and its 95% confidence interval $\left(\hat{\mu_{0_{\text{inf}}}},\hat{\mu_{0_{\text{sup}}}}\right)$ given by

$$\hat{\mu_{0_{\text{inf}}}}=\hat{\mu_{0}}-1.96\text{se}(\hat{\mu_{0}})$$

and

$$\hat{\mu_{0_{\text{sup}}}}=\mu_{0}+1.96\text{se}(\hat{\mu_{0}})$$

where $\text{se}(\hat{\mu_{0}})$ is the estimated standard error of the expected response given by the GAM evaluated at point $\hat{\mu_{0}}$.

Finally, we obtain the interval $\left(x_{0_{\text{inf}}},x_{0_{\text{sup}}}\right)$ by reversing the process, such that

$$f^{-1}(g(\hat{\mu_{0_{\text{inf}}}})-\alpha_{0})=x_{0_{\text{inf}}}$$

and

$$f^{-1}(g(\hat{\mu_{0_{\text{sup}}}})-\alpha_{0})=x_{0_{\text{sup}}}$$

This is, the points $x_{0_{\text{inf}}}$ and $x_{0_{\text{sup}}}$ are thus the cut points that determine the average-risk category. Therefore, the interpretation of this average-risk category, is that for any $x\in (x_{0_{\text{inf}}},x_{0_{\text{sup}}})$ this point is considered an average-risk point, the same as *x*_0_.

If *x*_0_ is not unique, i.e., if the graph displayed crosses the vertical axis more than once at point 0, then there will be more than one average-risk category, provided that the band at *x*_0_, is not too wide (with the band being taken to mean the confidence interval shown in the graph). In other words, if *x*_01_ and *x*_02_ are two values for which the graph crosses the vertical axis at point 0, two average-risk categories will be considered, as long as $\left(x_{01_{\text{inf}}},x_{01_{\text{sup}}}\right)$ and $\left(x_{02_{\text{inf}}},x_{02_{\text{sup}}}\right)$ do not overlap. If the last happens, we hypothesise that it may be due to two situations. The first is that one of the two intervals is based on a very small sample size which leads to a non-accurate and hence very wide interval. The second is the overlapping of two intervals of similar size. Under the first circumstance, we suggest to dismiss the interval based on a very small sample. However, if the second circumstance happens, we will consider the union of both intervals as the average-risk category.

**Figure 1:**
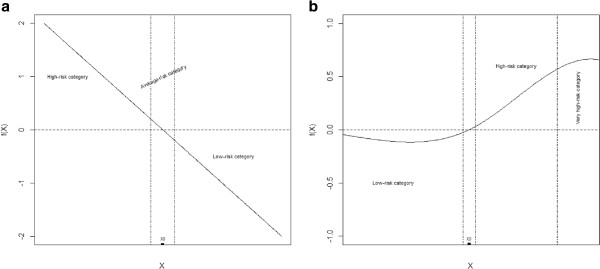
**Graphical representation of two hypothetical shapes between the predictor and outcome using generalised additive models. (a) **Linear relationship **(b) **Non-linear relationship.

Once the average-risk category has been defined, the following two possible scenarios are considered in order to create high- and low-risk categories: 

1. The relationship shown on the graph between the covariate and the outcome given by the GAM is linear along the entire range of *X*. Under this scenario, we propose to categorise *X* into a minimum of three categories, with the cut points for the three being selected as the limits of the average-risk category. This hypothetical situation is depicted in Figure 1(a). Moreover, if more categories are needed to ensure that the linear relationship between the covariate and the outcome is adequately retained, these could be created by considering appropriate cut points, preferably based on clinical criteria, in any of the designated high-risk or low-risk categories; or,

2. The relationship shown on the graph between the covariate and the outcome given by the GAM is not linear, which means that there is either a jump or a change in the slope. Firstly, we propose to proceed as described above for the first three categories, labelled "average-risk", "low-risk" and "high-risk". Secondly, the points at which the slope change occurs will be deemed to be extra cut points. Consequently, this will lead to the corresponding low-risk or high-risk categories, or both, being re-categorised as very low- and low- or very high- and high-risk categories respectively. The selection of these extra cut points will be made on the basis of graphical visualisation of the slope and the clinical significance of the cut point in question. This hypothetical situation of one extra cut point is depicted in Figure 1(b)

In both cases, the need for more than the proposed minimum number of categories will be evaluated by comparing the results of adding more categories to the original continuous covariate, using the validation criterion explained in detail below.

### Implementation methods

#### ***Application to the IRYSS-COPD study database***

We applied the methodology proposed in this paper to a prospective cohort of patients with chronic obstructive pulmonary disease (COPD), including for study purposes a sample of 2877 patients with exacerbated COPD attending the emergency departments (EDs) of 16 participating hospitals in Spain. Information was recorded: at the date on which patients were evaluated at the ED; at the date on which the decision was made to admit patients or discharge them home; and during follow-up after admission or discharge home. The main data collected were those relating to patients’ respiratory function (e.g., arterial blood gases, respiratory rate (RR), dyspnea) on arrival at the ED and at the time of the decision to admit them to ward or discharge them home (for fuller details see the IRYSS-COPD Study [16]).

By way of an illustration of the application of the proposed methodology, we selected part of the IRYSS-COPD Study, specifically: one dichotomous outcome; poor evolution in the first 7 days from arrival at the ED (which includes any of the following: death, ICU admission, the need for invasive mechanical ventilation, cardiac arrest, non-invasive mechanical ventilation for more than 2 days when mechanical ventilation was not needed before admission, and/or admission to an intermediate respiratory care unit for 2 or more days [16]); and two continuous covariates, namely, the blood gas parameter, PCO2, and the RR. Exacerbated COPD is a severe condition quite commonly seen at EDs, where proper decision-making tools are vitally important for performing the necessary diagnoses and implementing the treatments that are urgently required. Among the basic diagnosis tests used for classifying the severity of presentation of exacerbated COPD in such patients, arterial blood gases are the main tool. PCO2 is a highly valuable item of information drawn from arterial blood gases; similarly, another key item of information is proper assessment of patients’ respiratory rate, something that is invariably affected in these cases. Furthermore, these two variables represent the two possible theoretical scenarios described above.

Considering a dichotomous outcome Y, the link *logit* was used in the GAM model. In such a case, the model described in (2) for one covariate *X* would be more precisely specified by the following expression:

(3) $$\begin{array}{llll} \text{logit}(p)=\alpha_{0}+f(X) & & & \end{array}$$

where *p* represents the probability of *Y* = 1, which is interpreted as the probability of a patient having a poor evolution.

#### ***Validation***

The total sample was randomly divided into a derivation (60%) and validation (40%) sample. Cut points were obtained using the derivation sample while validation sample was used for method evaluation purposes.

The method for categorising continuous covariates was evaluated, by comparing the performance of the proposed categorical predictor in the model to that of the original continuous variable modelled by a GAM as the best option in the same model. In addition, we also compared the proposed categorisation to the dichotomised variable suggested by Hin et al. (1999) [8].

To compare models using different approaches to represent the same covariate, two criteria were selected: the first was the Akaike Information Criterion (AIC), a well-known, classical method for comparing two models [17]; the second method of evaluation was based on the specific model defined in equation (3) and the study’s designated purpose. In this particular case, we desired to evaluate the prediction ability of the model selected. We thus proposed to use the area under the receiver operating characteristic (ROC) curve (AUC) as the parameter that quantifies a logistic model’s ability to predict. The AUCs for two ROC curves were compared using the DeLong test [18].

Additionally, the goodness-of-fit of the proposed categorisation was evaluated by means of the Hosmer-Lemeshow test, which assesses the concordance between observed and expected event rates in a logistic regression model [19]. Finally, the need for additional categories, in excess of a minimum of three, was also checked by testing for statistically significant differences in risk between additional and adjacent categories.

Finally, we performed a sensitivity analysis in order to assess the impact sample size may have on the width of the average-risk category. We recalculated the average-risk category for PCO2 and for samples of size 200, 400, 600, 800, 1000 and 1200 obtained resampling without replacement from the original sample.

All statistical analyses were performed using the R software package. The mgcv, BB and pROC libraries were specifically used to compute the GAM model, cut points and AUC values respectively. The R code used to implement the proposed methodology in the application presented here is shown in Additional file 1.

## Results

### Categorisation process

**Figure 2:**
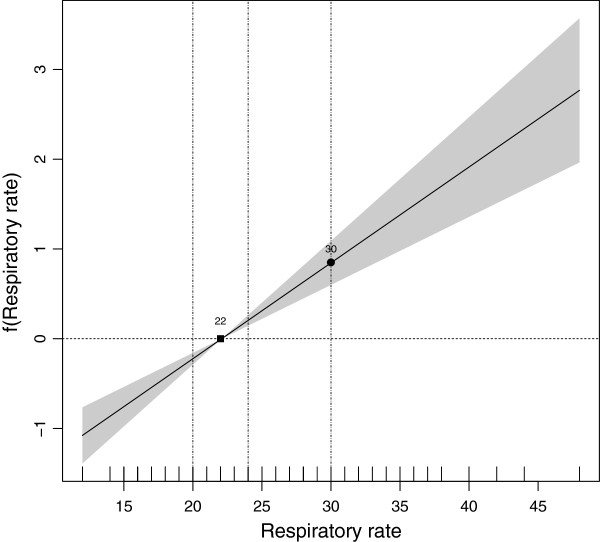
**Graphical representation of the cut points obtained for the respiratory rate. ** Cut points obtained, based on the relationship between respiratory rate and poor evolution.

**RR**: The relationship between the RR and poor evolution, as plotted by an additive logistic regression model with smoothing P-splines, is depicted in Figure 2. It will be seen that the relationship between the RR and poor evolution was linear and that there was only one value for which *f*(*x*_0_) = 0 (*x*_0_ = 22). Application of the proposed methodology to determine the limits of the average-risk category showed this category to be (20-24). It was therefore decided that the RR would be classified into 3 categories (Figure 2), with a high risk of poor evolution for values above 24 and a low risk of poor evolution for those below 20: accordingly, our final proposal for classifying the RR into three categories was ≤ 20; (20,24]; > 24.

In the search for an optimal fit to the original model, the need for a fourth category was explored. Taking the number of individuals with an RR above 24 and the available clinical information about the disease into consideration [20], an additional cut point of 30 was selected. The following four-category RR version, namely, ≤ 20; (20,24]; (24,30]; > 30, was thus also tested.

In addition, the RR variable was dichotomised as indicated before, whereby an RR value for which there is an average risk of poor evolution is taken as the cut point, which in our case was 22: consequently, our dichotomous RR proposal was ≤ 22; > 22.

**Figure 3:**
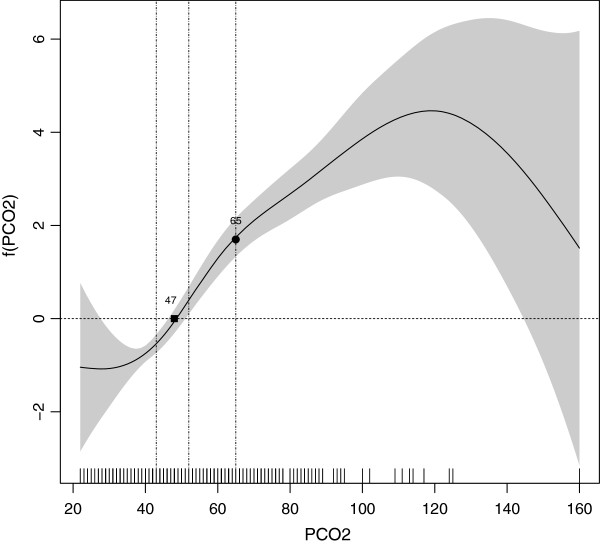
**Graphical representation of the cut points obtained for PCO2. **Cut points obtained, based on the relationship between PCO2 and poor evolution.

**PCO2**: The relationship between PCO2 and poor evolution, as plotted by an additive logistic regression model with smoothing P-splines, is shown in Figure 3. In this case, the relationship did not prove linear, with a trend towards a less steep slope for higher values. We started by calculating an average-risk category: this was (43-52), meaning that there was a high risk of poor evolution for values above 52 and a low risk of poor evolution for those below 43. The need to select more cut points was then explored. From 40 to 43, the relationship was linear, and below this there was no significance because the confidence interval was too wide. Above 52, however, there were several points where there was a slope change. While all values above 80 were dismissed, since the confidence interval was too wide and there were very few patients with PCO2 values as high as this, graphical examination of values below 80 nevertheless showed 65 to be a reasonable cut point for distinguishing between the high- and very high-risk categories. Finally, we decided on the 4-category PCO2 proposal shown in Figure 3, namely, ≤ 43;(43-52]; (52-65]; > 65.

As in the case of the RR above, the PCO2 variable was also dichotomised, whereby a PCO2 value for which there is an average risk of poor evolution is taken as the cut point. Since the value in this particular instance was 47, the dichotomous PCO2 proposal was therefore ≤ 47; > 47.

### ***Validation***

We considered the assessment parameters, AIC and AUC, obtained from the continuous predictor in a GAM as the best option and those obtained with the dichotomised option as perhaps needing improvement. Detailed results of the validation process are shown in Table 1.

**Table 1:** Categorisation of the respiratory rate (RR) and PCO2 covariates from the IRYSS-COPD study, based on the proposed methodology

	**Derivation ( *N = 805 *)**		**Validation ( *N = 545 *)**		
**Variable**	**Cut points**		**AIC**	**AUC**	p-value*
RR	Continuous‡		317.10	0.634	-
RR	Dichotomised	≤ 22	318.10	0.594	0.079
		> 22			
RR	3-category	≤ 20	314.50	0.638	0.8198
		(20-24]			
		> 24			
RR	4-category	≤ 20	316.2	0.640	0.6833
		(20-24]			
		(24-30]			
		> 30			
PCO2	Continuous‡		250.26	0.825	-
PCO2	Dichotomised	≤ 47	281.50	0.742	≤ .0001
		> 47			
PCO2	3-category	≤ 43	270.76	0.779	0.0002
		(43-52]			
		> 52			
PCO2	4-category	≤ 43	258.11	0.810	0.1148
		(43-52]			
		(52-65]			
		> 65			

Cut points were obtained in the derivation sample. AIC and AUC values were computed in the validation sample *Corresponding to the DeLong’s test for comparing the AUC of each model with the continuous option ‡AIC and AUC were calculated from the GAM

**RR**: Our approach proposed that the RR be classified into a minimum of 3 categories, for which the following values were obtained: AIC=314.5 and AUC=0.638 for the 3-category option versus AIC=317.1 and AUC=0.634 for the continuous predictor, with no statistically significant differences being found between the two AUCs (*p* = 0.8198). The respective values for the dichotomous predictor were AIC=318.1 and AUC=0.594. Statistically significant differences in AUCs were observed between the dichotomous and proposed 3-category approaches (*p* = 0.049).

Lastly, the 4-category option yielded an AIC of 316.2 and an AUC of 0.64. No statistically significant differences in AUCs were observed when this was compared to both the continuous (*p* = 0.6833) and the 3-category approaches (*p* = 0.5968). Moreover, when the model for the 4-category option was adjusted, however, non-statistical differences were found between the estimated parameters for the (24,30] and > 30 categories (*p* = 0.074). Detailed results of the adjusted model are shown in Table 2.

**Table 2:** **Results of the adjusted logistic regression models with the 4–category option for the respiratory rate (RR) and PCO2 covariates from the IRYSS-COPD Study, showing estimates of the beta coefficients, their 95 *% * confidence intervals and the p-values of their significance**

**Category**	**Estimate**	**95 *% *CI**	**p-value**
RR ≤ 20	-1.76	(-2.37, -1.16)	< 0.0001
RR (20-24]	-1.22	(-1.86, -0.58)	0.0002
RR (24-30]	-0.56	(-1.18, 0.06)	0.074
RR > 30	-	-	-
Hosmer-Lemeshow test p-value > 0.05			
PCO2 ≤ 43	-3.48	(-4.18, -2.86)	< 0.0001
PCO2 (43-52]	-2.62	(-3.27, -2.03)	< 0.0001
PCO2 (52-65]	-1.44	(-1.97, -0.93)	< 0.0001
PCO2 > 65	-	-	-
Hosmer-Lemeshow test p-value > 0.05			

Additionally, the models for the 4-category and 3-category options were both well calibrated (Hosmer-Lemeshow test p-values > 0.05 in both cases). Furthermore, when the proposed 3-category option was applied to other outcomes, such as admission to ward, it was able to detect differences between categories. When compared to the average-risk category, the estimates of the coefficients in a logistic model for the low- and high-risk categories were -0.81 and 0.68 respectively (p-values < 0.001 in both cases).

**PCO2**: Our approach proposed that the PCO2 variable be classified into 4 categories, for which the following values were obtained: AIC=258.1 and AUC=0.81 for the 4-category option versus AIC=250.26 and AUC=0.825 for the continuous predictor, with no statistically significant differences between the two AUCs (*p* = 0.1148). The respective values for the dichotomous predictor were AIC=281.5 and AUC=0.742. Statistically significant differences in AUCs were observed, not only between the continuous and dichotomous approaches (*p* < 0.0001), but also between the dichotomous and proposed 4-category approaches (*p* = 0.0001). In addition, we verified the need to create a fourth category, by comparing the 4-category against the 3-category predictor (our minimum proposal). Statistically significant differences were found between both AUCs (*p* = 0.0004), with the AUC value for the 3-category predictor being 0.779.

Furthermore, when the model for the 4-category option was adjusted, statistically significant differences were found between the estimated parameters for the (52-65] and > 65 categories (*p* < 0.0001). Lastly, the Hosmer-Lemeshow test assessed the goodness-of-fit of both the 3- and 4-category options (*p* > 0.05). Detailed results are shown in Table 2.

Moreover, when the proposed 4-category option was applied to other outcomes, such as admission to ward, it was able to detect differences between categories. When compared to the average-risk category, the estimates of the coefficients for the low-, high- and very high-risk categories were -0.16, 0.67 and 1.49 respectively (p-values of 0.24, < 0.001 and < 0.001 respectively).

Additionally, testing the performance of both variables in a multivariate logistic regression model, comparison between the model with RR and PCO2 as continuous and that with these two variables classified into 3 and 4 categories respectively yielded no statistically significant differences in AUCs (AUC=0.827 for the former versus AUC=0.814 for the latter; *p* = 0.7021).

**Figure 4:**
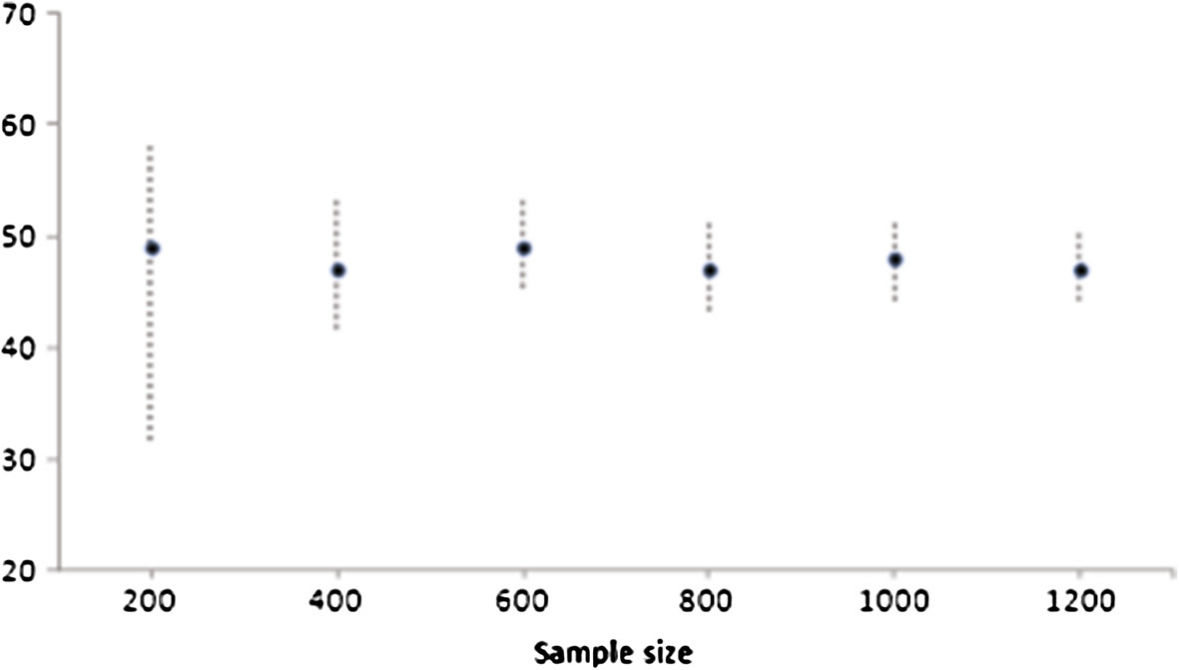
Graphical representation of the average-risk category width and location for PCO2 based on sample size.

Finally, results from the sensitivity analysis for sample size variation for PCO2 are shown in Figure 4.

## Discussion

In clinical practice, decisions need to be made by reference to clinical parameters, which are usually continuous measurements. Accurate knowledge of the relationship between such parameters and the risk of developing a certain outcome helps identify individuals most at risk. Considering these parameters as continuous predictors is preferable from a statistical point of view, since categorisation may lead to loss of information and reduction in power [3]. Nevertheless, in the development of CPRs for application in clinical practice, it may be preferable for a certain amount of information to be sacrificed in the interests of enhanced utility and ease of use in daily clinical practice.

When the researchers were developing a prediction model in the context of the IRYSS-COPD Study, several reasons led them to categorise the continuous clinical variables that had been collected for study purposes. Not all the data needed for the study were available in the clinical records, and even where these were available, they were not always in a desirable format, appearing, for instance, as a description of patient status rather than a numerical value, e.g., in the IRYSS-COPD Study, some patients’ RR was recorded as “eupneic” or “taquibneic” instead of being cited as a number on a continuous scale. Despite the fact that clinicians failed to agree on the cut points to apply to each code, they did, in contrast, regard the “eupneic” and “taquibneic” patients as “normal” and “altered” respectively, which means that, while they would be able to classify such patients on a categorical scale, they would nevertheless leave them as missing data on a continuous scale. The adoption of categorisation of these parameters that are partially available as continuous variable and partially available as ordinal variable facilitate reconciliation of information. Moreover, in a study such as the IRYSS-COPD [16], ED clinical practice prevails over research requirements. Hence, the data available was the information routinely recorded at the ED for COPD patients. In our opinion, the sacrifice of some information in the subset of data recorded as continuous variable to avoid information exclusion of the subset of data recorded as ordinal variable is a worthwhile trade-off. One consequence of this was that, categorisation of variables such as RR, enabled an average of 38% of values not to be excluded.

Bearing this in mind, our aim was to furnish a method of categorisation for clinical parameters selected as predictors, taking the following three principles into account: 1) categorisation would depend on the outcome of interest and, by extension, on the model selected for analysis; 2) any loss of information as compared to the continuous predictor would be minimal; and 3) the method would provide clinicians with a convenient and easily interpretable categorical predictor.

Studying the relationship between the predictor and the outcome was absolutely necessary in order to fulfil the first two principles. We decided to start by plotting the relationship graphically. GAM functions were selected because they are a powerful technique for estimating the relationship between continuous predictor variables and outcomes [11], with no need for any assumptions about this relationship. P-spline smoothers are suggested in the literature as being the most convenient technique for estimating smooth functions [21]. When developing the proposed methodology, we considered the method suggested by Hin et al. (1999) [8] as the first approach to our designated objective: their proposal consists of dichotomising the predictor variable by using GAMs, taking the value for which an average risk is obtained as the cut point. We felt that a specific cut point could be highly dependent on the sample size or even the random sample itself, and that an interval, rather than a single point, might therefore be a wiser choice as an average-risk category. Although sample size would also affect the length of any interval, the latter would nevertheless provide more information to clinicians than would a single point. On the other hand, ensuring a minimum loss of information vis-à-vis the continuous variable was one of our stated goals, and so we hypothesised that two categories were possibly not enough.

Our proposal, motivated in part by the work by Hin et al (1999) [8], occupies the middle ground between their approach and the original continuous predictor. This paper shows that our categorisation proposal does not lose critical information from the original predictor, respects the relationship between the original predictor and the outcome, and offers validated results with better predictive ability than does the dichotomous approach. Moreover, our proposal starts by suggesting a minimum number of 3 categories, and offering the necessary cut points to ensure that such a categorisation is a good categorised approximation to the continuous option. We have shown that in general, this approach improves Hin et al proposal (1999) in terms of fitting and prediction. The proposal includes a method to build an interval around the average-risk point using the inverse of the 95% confidence interval for the expected response. Although more complex techniques could provide other alternatives, in our opinion this is a simple and easy to understand way that shows the advantages and usefulness of a three category approach. In any given case, the need for more categories can be evaluated by researchers, depending upon the relationship between the predictor and the outcome, sample size and clinical knowledge of the problem. Moreover, any improvement resulting from the addition of more categories can be statistically tested. Although this is an illustrative example, in the application presented here we selected 4 categories for PCO2 and 3 categories for RR.

The first limitation of the proposed categorisation lies in the fact that it depends on the outcome and so its use cannot be recommended in every situation. This means that one might obtain different categorisation proposals for the same predictor, if one were to consider different outcomes or different modelling approaches. Although this characteristic of the proposal could be seen as a strength in the specific modelling situation, it must however be carefully reviewed when different modelling situations are being considered. Moreover, the proposed method of categorisation for one continuous predictor can be applied with additional (continuous or categorical) covariates in the model, which means that categorisation can be tailored to the model, including adjustment for several confounders. We have previously mentioned that the width of the average-risk category will depend on sample size, which is an obvious limitation. Sensitivity analysis showed that for sample sizes above 200 results were quite stable, while for size 200 the interval was much wider. In our opinion, for a moderate sample size as 200 probably there was not enough data to catch the relationship between the predictor and the response variable, and so, the average-risk category became very wide. In a simulation study with samples of size 200, we obtained that in 90% of them there were no differences between our proposal and the method suggested by Hin et al. (1999) [8]. Therefore, in this case we would recommend to check the performance of the dichotomised option first, merely for simplicity. Nevertheless, our proposal includes assessing the need for that third category in each case, and it compares the two versus the three categories approaches. The third limitation of our proposal resides in the subjectivity implied in the selection of extra cut points, in cases where more than three categories were necessary. We have given an outline of a way to do this in two different situations; and indeed, the addition of extra cut points in one of the specific applications was shown to improve the final result. However, we have also shown that improvement is progressive, increasing as more cut points are added, basically because comparison is made with the continuous predictor. Cut points are selected on the basis of the predictor/outcome relationship given by the graphical display, which means that as more cut points are added, not only will the categorical and the continuous predictors be more similar, but the selected categorisation will also be more data-dependent. Apart from statistical significance, therefore, an important part of researchers’ work will be to seek a balance between loss of information and practicality.

In brief, we propose a method for categorising continuous predictors in clinical prediction models, which includes both the number of categories and the best cut points. The proposal has been face-validated by clinicians, and the proposed categorical predictor has been shown to perform as successfully as the original continuous predictor when it comes to developing CPRs.

## Appendix 1: Splines

In general, the generalized additive model has the following structure

(4) $$\begin{array}{llll} \;g(\mu )=\alpha_{0}+\overset{p}{\underset{i=1}{\sum }}\;f_{i}\;(X_{i}) & & & \end{array}$$

where *μ* = *E*(*Y*) for *Y*, a response variable with some exponential family distribution, *g* is the *link* function, and *f*_*i*_ are some smooth functions of the covariates *X*_*i*_ for each *i* = 1,…,*p*.

The most common alternative for the estimation of the smooth functions *f*_*i*_ are based on splines. Splines are piecewise polynomials, being pieces defined by a sequence of knots *ζ*_1_ < *ζ*_2_ < …*ζ*_*m*_, in such a way that pieces join smoothly at these knots. A spline of degree *r* can be defined by a power series as follows:

(5) $$\begin{array}{llll} S(x)=\overset{r}{\underset{j=0}{\sum }}\alpha_{j}x^{j}+\overset{m}{\underset{k=1}{\sum }}\gamma_{k}{\left(x-\zeta_{k}\right)}_{+}^{r}, & & & \end{array}$$

where

$${\left(x-\zeta_{k}\right)}_{+}^{r}=\left\{\begin{array}{ll} x-\zeta_{k} & \text{if}x>\zeta_{k}\\ 0 & \text{otherwise} \end{array}\right)$$

The most popular splines are cubic splines or splines of degree 3 (*r* = 3), of the form:

(6) $$\begin{array}{llll} S(x)=\alpha_{0}+\alpha_{1}x+\alpha_{2}x^{2}+\alpha_{3}x^{3}+\overset{m}{\underset{k=1}{\sum }}\gamma_{k}{\left(x-\zeta_{k}\right)}_{+}^{3} & & & \end{array}$$

Smoothing splines are discussed in detail in Green and Silverman (1994) [13]. The use of smoothing splines with as many nodes as observations per GAM component, reduces the problem to the GLM with a penalty parameter of the curvature of the curve, thus imposing smoothing. Traditionally, smooth functions estimation is performed at a time, through an iterative algorithm on each smooth function, keeping the rest of the GAM components fixed in the obtained estimation, until the convergence is achieved. The resulting system of equations has *N* × *p* equations, which makes it a computationally difficult estimation problem (*N* number of observations and *p* number of covariates).

An alternative which allows to adjust all the smooth functions integrated in the GAM simultaneously is the use of regression splines, where the smooth functions are built as the sum of B-splines [22]. Actually, what it is used is a base of splines to build the smooth functions, hence the name B-splines. In general, a B-spline basis of degree *r* comprises: 

1. (*r* + 1) piecewise polynomials, each of degree *r*

2. These (*r* + 1) piecewise polynomials join at *r* inner knots.

3. At the junction point, the derivatives up to *r* - 1 order are continuous.

4. The B-spline is positive on a domain covered by *r* + 2 knots and zero otherwise.

5. Except at the frontiers, each B-spline overlaps with 2*r* neighbour piecewise polynomials.

6. For each value *x* of *X*, there are *r* + 1 non-zero B-splines.

In this way, a smooth B-spline base is independent of the response variable. The smooth functions are modelled as a base of B-splines depending on the following factors: i) the range of the independent variable *X*; ii) The number and location of knots; and iii) the degree of the B-spline.

When using this smoothing approach, for each GAM component, the smooth function is reduced to a linear combination of B-splines *f*_*i*_ = **B**_*i*_***α***_*i*_, where for each *i* = 1,…,*p*, **B**_*i*_ is the B-splines matrix of *N* × *m* dimension (*N* number of observations and *m* number of knots). This is,

$${\text{B}}_{i}=\left(\begin{array}{lll} B_{1i}(x_{1}) & \cdots & B_{\text{mi}}(x_{1})\\ \;\vdots & \;\vdots \\ B_{1i}(x_{N}) & \cdots & B_{\text{mi}}(x_{N}) \end{array}\right)$$

and ***α***_*i*_ = (*α*_1*i*_,…,*α*_*mi*_)^**T**^ is the *m*-dimensional coefficient vector, associated to the B-spline basis, also called the B-splines amplitudes. As an example, in the particular case in which there is one covariate and a normally distributed response variable, the expression (4) reduces to:

(7) $$\begin{array}{llll} \alpha_{0}+\overset{m}{\underset{k=1}{\sum }}B_{k}(x)\alpha_{k} & & & \end{array}$$

so that ***α*** can be estimated as in a GLM model by minimizing the residual sum of square given by:

(8) $$\begin{array}{llll} \overset{N}{\underset{j=1}{\sum }}{\left(y_{j}-\overset{m}{\underset{k=1}{\sum }}b_{\text{jk}}\alpha_{k}\right)}^{2}, & & & \end{array}$$

where *b*_*jk*_ = *B*_*k*_(*x*_*j*_) is the value of the *k*-th B-spline at the point *x*_*j*_. The smoothness of the curve depends on the number of B-splines, hence, the number of knots and the value of the amplitudes, and therefore, the ***α***vector. The advantage of this option is that B-splines are easy to build, but the main problem now resides in the optimization of the position and the number of knots.

An intermediate alternative to build the smooth functions, which considers both the smoothing splines and the regression splines advantages is the use of penalized splines also known as P-splines and introduced by Eilers and Marx [15]. P-splines use fewer knots than smoothing splines and introduce more general roughness penalties which relaxes the importance of the knot location. The idea is to start with a B-spline basis, considering more knots than if we were using regression splines (but not as many as observations), and subsequently introduce a penalization in the same way as if we were using smoothing splines. However, in P-splines, the coefficients of the model are the ones penalized rather than the curvature. In this way, similar coefficients are obtained for those nearby, and this provides a computational advantage over smoothing splines.

The substitution of the term B-spline for the term P-spline is motivated by the fact that the model fitting method is modified. The model based on P-splines is based on setting a reasonable number of knots, adding a penalty to the differences in coefficients between each two adjacent knots. Thus the number of knots ensures flexibility while avoiding the over-estimation and ensuring smoothness by penalization.

If we consider the same conditions as in (4) the GAM based on P-splines can be expressed as:

(9) $$\begin{array}{llll} g(\mu )=\alpha_{0}+\text{B}\alpha & & & \end{array}$$

where ***α*** = ${\left(\alpha_{11},\ldots ,\alpha_{1m_{1}},\ldots ,\alpha_{p1},\ldots ,\alpha_{pm_{p}}\right)}^{\text{T}}$ is the coefficients vector, *m*_*i*_ is the number of P-splines, this is, the number of knots, for the *i*^*th*^ covariate, for each *i* = 1,…,*p*. and **B** is the $N\times (1+\overset{p}{\underset{i=1}{\sum }}m_{i})$ regressor matrix defined as

$$\left(\begin{array}{lllllll} B_{11}(x_{11}) & \cdots & B_{1m_{1}}(x_{11}) & \cdots & B_{p1}(x_{1p}) & \cdots & B_{pm_{p}}(x_{1p})\\ \;\vdots & \cdots & \;\vdots & \cdots & \;\vdots & \cdots & \;\vdots \\ B_{11}(x_{N1}) & \cdots & B_{1m_{1}}(x_{N1}) & \cdots & B_{p1}(x_{\text{Np}}) & \cdots & B_{pm_{p}}(x_{\text{Np}}) \end{array}\right)$$

In general, the estimation method consists on maximizing the penalized version of the log-likelihood expressed as:

(10) $$\begin{array}{llll} l^{*}=l(y;\alpha )-\frac{1}{2}\overset{p}{\underset{i=1}{\sum }}\lambda_{i}\alpha_{i}^{T}{\text{P}}_{i}\alpha_{i} & & & \end{array}$$

where the term *l*(*y*;***α***) represents the log-likelihood of the vector of response variables Y; for each *i*=1,…,*p* the *λ*_*i*_ ≥ 0 are the smoothing parameters and **P**_*i*_ is a *m*_*i*_ × *m*_*i*_ dimension matrix that defines the penalty structure over the *d*-dimensional differences between every two adjacent P-splines coefficients. The estimation method is explained in detail in [23].

## Abbreviations

AIC: Akaike Information Criterion; : ; COPD: Chronic Obstructive Pulmonary Disease; CPR: Clinical Prediction Rule; ED: Emergency Department; GAM: Generalised Additive Model; GLM: Generalised Linear Model; ROC: Receiver Operating Characteristic; RR: Respiratory Rate; PCO2, partial pressure of carbon dioxide in the blood; CI: Confidence Interval.

## Competing interests

The authors declare that they have no competing interests.

## Authors’ contributions

Study design, IB, IA and JMQ; proposal of analysis to be performed, IB and IA; statistical analysis and outcomes: IB and IA; interpretation of results, IB, IA and JMQ; drafting, review and revision of text, IB, IA and JMQ; data-collection, all IRYSS-COPD Group members. All authors read and approved the final manuscript.

## Pre-publication history

The pre-publication history for this paper can be accessed here:

http://www.biomedcentral.com/1471-2288/13/83/prepub

## Supplementary Material

Additional file 1 Appendix 2: R code. Click here for file
