# Supplementary material for: Use of generalised additive models to categorise continuous variables in clinical prediction
Source: BMC Med Res Methodol. 2013 Jun 26;13:83. doi: 10.1186/1471-2288-13-83 (PMC3716996; doi:10.1186/1471-2288-13-83)
Supplement: Additional file 1 — Appendix 2: R code. [file 1471-2288-13-83-S1.pdf]

Appendix 2: R code

```
library(pROC)
library(mgcv)
library(BB)

######################
# Respiratory Rate
#####################
# Fit the GAM model and plot the graph
fitted.model.rr <- gam(Y~s(X.rr, bs="ps"), method="REML", family=binomial)
plot(fitted.model.rr,shade=T,scale=0,
     xlab="Respiratory rate", ylab="f(Respiratory rate)")
abline(h=0,lty=2,lwd=0.5)

#### Look for x0, such that f(x0)=0
f.smooth <- function (x, model, p0) {
               df <- data.frame(x)
               names(df) <- attributes(terms(model))$term.labels
               res <- as.numeric(predict(model, newdata=df, type="terms")
                                 - family(model)$linkfun(p0) + coef(model)[1])
               res
}
f.smooth2 <- function (x, model, p0) {
               df <- data.frame(x)
               names(df) <- attributes(terms(model))$term.labels
               res <- as.numeric(predict(model, newdata=df, type="terms"))
               res
}
x0_rr <-dfsane(par=20, fn=f.smooth2, model= fitted.model.fr,
                p0=0, control=list(trace=FALSE))

df<-data.frame(X.rr=22)
logit_p<-predict(fitted.model.rr, newdata=df)
p0<-exp(logit_p)/(1+exp(logit_p))
pred<-predict(fitted.model.rr, se=TRUE, newdata=df)
u.ci <- exp(pred$fit + 2*pred$se.fit)/(1+exp(pred$fit + 2*pred$se.fit))
l.ci <- exp(pred$fit - 2*pred$se.fit)/(1+exp(pred$fit - 2*pred$se.fit))
inf_x0<-dfsane(par=seq(18,22,l=1350), fn=f.smooth,
                model= fitted.model.fr, p0=l.ci, control=list(trace=FALSE))
sup_x0<-dfsane(par=seq(22,30,l=1350), fn=f.smooth,
                model= fitted.model.fr, p0=u.ci, control=list(trace=FALSE))

## Create the categorised variable. Compute AUC and AIC and compare AUCs
rr.cut.4<-cut(X.rr,c(20,24,30),include.lowest=TRUE,right=TRUE )
rr.cut.3<-cut(X.rr,c(20,24),include.lowest=TRUE,right=TRUE )
rr.cut.2<-cut(X.rr,c(22),include.lowest=TRUE,right=TRUE )
fit.rr.cut.4<-glm(Y~rr.cut.4,family=binomial)
fit.rr.cut.3<-glm(Y~rr.cut.3,family=binomial)
fit.rr.cut.2<-glm(Y~rr.cut.2,family=binomial)
roc.rr.4<-roc(Y,fit.rr.cut.4$fitted)
roc.rr.3<-roc(Y,fit.rr.cut.3$fitted)
roc.rr.2<-roc(Y,fit.rr.cut.2$fitted)
roc.rr.gam<-roc(Y,fitted.model.rr$fitted)
roc.test(roc.rr.4,roc.rr.gam)
roc.test(roc.rr.3,roc.rr.gam)
roc.test(roc.rr.2,roc.rr.gam)
roc.test(roc.rr.4,roc.rr.3)
```
